# How did governmental interventions affect the spread of COVID-19 in European countries?

**DOI:** 10.1186/s12889-021-10257-2

**Published:** 2021-02-26

**Authors:** Richard A. J. Post, Marta Regis, Zhuozhao Zhan, Edwin R. van den Heuvel

**Affiliations:** 1 Department of Mathematics and Computer science, Eindhoven University of Technology, P.O. Box 513, 5600 MB Eindhoven, The Netherlands; 2 Department of Preventive Medicine and Epidemiology, School of Medicine, Boston University, Boston, MA 02118 USA

**Keywords:** Effective-contact rate, COVID-19, Governmental interventions, Social distancing, Epidemic disease modeling

## Abstract

**Background:**

To reduce the transmission of the severe acute respiratory syndrome coronavirus 2 in its first wave, European governments have implemented successive measures to encourage social distancing. However, it remained unclear how effectively measures reduced the spread of the virus. We examined how the effective-contact rate (ECR), the mean number of daily contacts for an infectious individual to transmit the virus, among European citizens evolved during this wave over the period with implemented measures, disregarding a priori information on governmental measures.

**Methods:**

We developed a data-oriented approach that is based on an extended Susceptible-Exposed-Infectious-Removed (SEIR) model. Using the available data on the confirmed numbers of infections and hospitalizations, we first estimated the daily total number of infectious-, exposed- and susceptible individuals and subsequently estimated the ECR with an iterative Poisson regression model. We then compared change points in the daily ECRs to the moments of the governmental measures.

**Results:**

The change points in the daily ECRs were found to align with the implementation of governmental interventions. At the end of the considered time-window, we found similar ECRs for Italy (0.29), Spain (0.24), and Germany (0.27), while the ECR in the Netherlands (0.34), Belgium (0.35) and the UK (0.37) were somewhat higher. The highest ECR was found for Sweden (0.45).

**Conclusions:**

There seemed to be an immediate effect of banning events and closing schools, typically among the first measures taken by the governments. The effect of additionally closing bars and restaurants seemed limited. For most countries a somewhat delayed effect of the full lockdown was observed, and the ECR after a full lockdown was not necessarily lower than an ECR after (only) a gathering ban.

**Supplementary Information:**

The online version contains supplementary material available at 10.1186/s12889-021-10257-2.

## Background

To reduce the transmission of the severe acute respiratory syndrome coronavirus 2 (SARS-CoV-2) in its first wave (i.e., the period February to May in 2020), European governments have implemented several non-pharmaceutical interventions aimed at reducing the number of contacts among individuals [1]. The implementation of these governmental measures differed per country, but they followed a similar pattern (Appendix A). Events involving large numbers of participants were first suspended. Then the schools were closed, shortly followed by closure of non-essential services like bars and restaurants. Finally, gathering was banned (Netherlands and Germany) or citizens were forced to stay home (United Kingdom (UK), Italy, Spain and Belgium). The latter intervention was often followed up by further restrictions, e.g. stricter surveillance by authorities. While in most countries these policies were applied to the entire nation, Italy and Spain started to apply these measures locally in the so-called ‘red zones’ (regions where the spread started).

Late March 2020, stabilization of the number of daily new cases, deaths and hospitalizations was observed after the implementation of the governmental measures. These effects have been quantified in country-specific studies in which the estimated reproduction number was compared before, during and after measures were taken [2, 3]. Measure-specific effects on the transmission in the period February to March 2020 have also been estimated for some European countries [1]. Those authors analyzed the observed deaths and claimed that ordering of lockdown, closure of schools, ban on public events and encouragement of social distancing would reduce the reproduction number by approximately 50, 20, 10 and 10%, respectively. Their work assumed that the relative improvement for these interventions was the same across countries, and that measures had an immediate and constant effect (thus excluding possible delays). Another study looking at the same period used the number of newly confirmed infections, and showed that the effect of venue closure, gathering ban, border closure, work ban, public event ban, closure of schools and an additional lockdown reduced the reproduction number by approximately 36, 34, 31, 31, 23, 8 and 5%, respectively. They also assumed homogeneous reductions across countries and incorporated a fixed delay of 7 days before an effect would be visible in the number of new infections [4].

Measure-specific estimates of the reduction of the virus spread differ among these two studies [1, 4]. This is not surprising, since estimation of the influence of governmental interventions is hindered by several serious limitations. Firstly, interventions may have had a partially delayed effect and it is difficult to identify how the effect of a measure changed over time. Restricting changes in the contact-rate profiles to the moments of interventions may lead to some false conclusions. Alternatively, change-point models have been used to study the number and moments in time of changes in the spreading rate [5]. However, this approach does not overcome the second limitation: the incompleteness of data. Only a fraction of the infectious people were tested such that the real number of infectious individuals was largely unknown. Also the ‘recovery’ of non-hospitalized contagious individuals was mostly non-recorded. Both are important elements in the estimation of epidemic spread models such as the Susceptible-Exposed-Infected-Recovered (SEIR) model, to be able to determine (time-varying) contact rates and the reproduction number [6, 7]. The third limitation concerns the transmission times that are commonly assumed to be exponential distributed in epidemic disease models [6], while for Coronavirus Disease 2019 (COVID-19) it was shown that the incubation period is better fitted by a Weibull distribution [8–12].

To accommodate these three limitations for understanding change in transmission of COVID-19 in the first wave, we have relaxed some assumptions of the SEIR model and implemented a data-driven sequential approach to estimate time-dependent contact-rate profiles, disregarding a priori information on governmental measures. We were then able to compare the change points in the contact-rate profiles to the moments at which measures were implemented. This way, for each country separately, we observed whether the influence of measures was visible, immediate or delayed. The advantage of our stratified country-specific analysis is that it does not require harmonization of governmental interventions across countries, it does not impose similarity across countries (e.g. decrease in the contact rate) and it allows a flexible measure-delay effect per country rather than imposing a fixed one. We illustrate our results for seven European countries that adopted different strategies.

## Methods

### Data collection

Daily counts of confirmed infections in the period February to April were obtained from the online interactive dashboard hosted by the Johns Hopkins University [13]. These numbers were cross-validated with other sources (official sites of authorities and https://www.worldometers.info/coronavirus/). The hospitalization numbers were extracted from the official daily reports released by the public health authorities separately for each country (Table S2). Data collected on recoveries is largely incomplete and was therefore not used in this study. We collected data until 2020-04-09, that includes information before, during, and after governmental interventions in the first wave, but does exclude relaxation of interventions. Additionally, we used data on the mobility of communities made available by Google [14] (Figs. S9-S15).

### Epidemic disease model

**Fig. 1:**
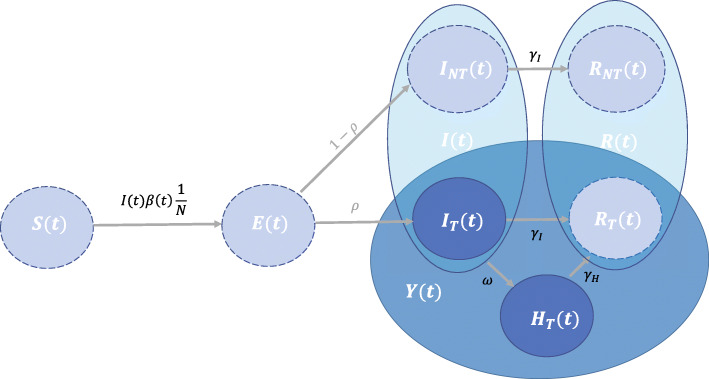
Schematic representation of the SEIR model. When there are both susceptible individuals, *S*(*t*), as well as infectious individuals, *I*(*t*), a susceptible individual gets exposed *E*(*t*) at an exponential rate of *β*(*t*)*S*(*t*)/*N*. Once exposed it takes a Weibull distributed time before the individual becomes infectious. The individual is tested with probability *ρ*, *I*(*t*), and might subsequently be hospitalized, *H*(*t*)). Infectious individuals that will not be tested, *I*_*NT*_(*t*), cannot be hospitalized. The infectious period of non-hospitalized infectious individuals is assumed to follow an Exponential distribution after which the individuals are transferred to the removed states

We modelled the daily confirmed numbers of infections, hospitalizations, and deaths per country with an extended and time-discretized SEIR model [6, 15] (Fig. 1), such that the total population, of size *N*, can be distributed over the Susceptible (S), Exposed (E), Infectious (I) and Removed (R) states (Fig. 1). We assumed that the entire population was susceptible, as reported by the World Health Organization [16].

A susceptible individual in *S*(*t*) can become exposed (*E*(*t*)) after an effective contact with an infectious individual. There will be an incubation period of random length before individuals become infectious and enter state *I*(*t*). We assumed that this delay follows a Weibull distribution, with a shape parameter equal to 2.32 and a scale parameter equal to 6.50 (giving an average delay of 5.76 days), based on the preliminary study of 33 COVID-19 patients [10]. A fraction *ρ* of these infectious individuals was tested, *I*_*T*_(*t*), while the number of non-tested infected individuals, *I*_*NT*_(*t*), was hidden (with *I*(*t*) = *I*_*T*_(*t*) + *I*_*NT*_(*t*)).

The removed state *R*(*t*), *R*(*t*) = *R*_*T*_(*t*) + *R*_*NT*_(*t*), refers to individuals that are no longer contagious (due to death or recovery). The number of susceptible and exposed individuals at day *t* was unknown. The number of removed people was also only partially known, since infectious individuals were only followed-up when they were hospitalized or when they died from the disease.

Furthermore, the infectious period of infected individuals (either tested or not) was unknown, so we assumed this period to be random, following an exponential distribution with a mean, ${\gamma}_I^{-1}$, of 2.3 days [17, 18]. The available information at day *t* included the observed cumulative number of confirmed infected individuals, *Y*(*t*) = *I*_*T*_(*t*) + *H*_*T*_(*t*) + *R*_*T*_(*t*), and the daily number of new hospitalizations, ∆*H*^+^(*t*), representing individuals that transited from *I*_*T*_(*t*) to *H*_*T*_(*t*). Since the number of hospitalizations was known, we did not use the transition rates *γ*_*H*_ and *ω* in this study. The balance equations for the transitions in the system presented in Fig. 1 can be found in Appendix B.

The main parameter of interest was the effective-contact rate (ECR), *β*(*t*), also known as the transmission rate. The ECR can be interpreted as follows: The a priori probability that an infectious person upon contact meets a susceptible individual is *S*(*t*)/*N*. If we assume that every contact between an infectious and a susceptible individual results in the transmission of the virus, then we can view *β*(*t*) as the average number of contacts on day *t*. In this case, the expected number of newly exposed individuals that one infectious person will introduce is *β*(*t*)*S*(*t*)/*N*. Since there are *I*(*t*) infectious individuals on day *t*, *I*(*t*)*β*(*t*)*S*(*t*)/*N* newly exposed individuals are expected on the same day. If we do not assume that all of the contacts lead to the transmission of the virus, then *β*(*t*) can be viewed as an effective number of contacts that leads to newly exposed individuals. The ECR was assumed to be time-dependent, since the introduction of different governmental interventions over time may affect the total number of contacts. The effective reproduction number *R*_*e*_(*t*) is commonly used to quantify the speed of transmission and is a direct function of *β*(*t*) and the recovery rate *γ*_*I*_ [19, 20], see the mathematical expression in Appendix C. During every day of the infectious period, starting at *t*, an infectious individual is expected to infect *β*(*t*) new individuals. Since the recovery rate *γ*_*I*_ is not influenced by governmental interventions, and not easy to estimate from the available data studied here, *β*(*t*) is the quantity of interest when evaluating the effect of interventions. For completeness we also report the estimates of *R*_*e*_(*t*) in Appendix C for the assumed value of *γ*_*I*_. Note that our main results in this research are insensitive to the choice of *γ*_*I*_ due to our approximation of *I*(*t*) over time, as described in the next section.

### Parameter estimation

Statistical inference in SEIR models with latent *S*(*t*) and *E*(*t*) classes have been well studied under the assumption that *I*(*t*) is known [21]. Furthermore, it is generally assumed that there are only a small number of changes in the ECR.

In order to obtain an ECR profile per country, we performed a stratified analysis in which we estimated *β*(*t*) at day *t* ∈ {1, 2, …, *n*}. We did not account for demographic characteristics but focused on the aggregated value for different countries separately. We allowed *β*(*t*) to differ every day, but since ECR estimates of subsequent days are highly correlated we imposed the restriction that the ECR profile is non-increasing: $\beta (t)={\sum}_{i=t}^n\exp \left({\alpha}_i\right)$, with *α*_*i*_ an unknown parameter. Based on the balance equations presented in Appendix B, we estimated the ECR from the following set of equations:

1 $$ \mathbbm{E}\left[\Delta  Y(t)\right]=\rho {\sum}_{i=0}^t{p}_{t-i}\cdotp \mathbbm{E}\left[{\Delta E}^{+}(i)\right]={\sum}_{i=0}^t{p}_{t-i}\cdotp \rho \cdotp I(i)\cdotp \beta (i)\cdotp S(i)/N, $$

where Δ is the symbol for daily change in the values, i.e. ∆*U*(*t*) = *U*(*t*) − *U*(*t* − 1), *E*^+^(*t*) = *E*(*t*) + *Y*(*t*) + *I*_*NT*_(*t*) + *R*_*NT*_(*t*), *ρ* the fraction of tested infected individuals, and *p*_*t*_ is the discretized Weibull probability that a susceptible individual becomes infectious *t* days after the beginning of the exposure. In words, the expected daily increase in reported cases equals a fraction *ρ* (as these are tested) of a weighted average of the individuals that were exposed in the days before. It is important to notice that the delay between onset of infection and symptoms was thus directly accounted for. Before we could estimate *β*(*t*) based on eq. (1) we should approximate i). the daily number of susceptible individuals *S*(*t*) and ii). the daily number of infectious individuals *I*(*t*) as these were both unknown and needed to be calculated from the observed data *Y*(*t*) (or ∆*Y*(*t*)) and ∆*H*^+^(*t*).

By adding up (1) over *t* and rearranging the elements of the totals we found the expected value of the total reported cases by day *t*,

2 $$ \mathbbm{E}\left[Y(t)\right]={\sum}_{i=1}^t\mathbbm{E}\left[\Delta  Y(i)\right]={\sum}_{i=1}^t\rho \left(\mathbbm{E}\left[\Delta  {E}^{+}(i)\right]\bullet {\sum}_{j=i}^t{p}_{t-j}\right). $$

Assuming that the Weibull probability was almost zero for the first 24 h (which fitted with the reported results [10]) and that *ρ* is known, the daily expected new exposures Δ*E*^+^(*t*) were iteratively calculated from (2), with *ρ*Δ*E*^+^(1) = Δ*Y*(2)/*p*_1_ and *ρΔE*^+^(2) = (Δ*Y*(3) − *ρ*Δ*E*^+^(1)*p*_2_)/*p*_1_. To stabilize these calculations, we first smoothed the daily cumulative number of confirmed infections *Y*(*t*) using a three-parameter logistic growth curve [22]. Given the estimates of the daily number of newly exposed individuals, we approximated the daily number of susceptible individuals *S*(*t*), assuming that the total population is susceptible [16], namely *S*(*t*) = *N* − *E*^+^(*t*).

To approximate *I*(*t*), we assumed that the hospitalized individuals, *H*_*T*_(*t*), could no longer infect others because they were quarantined. Then, the number of tested infectious individuals *I*_*T*_(*t*) at day *t* were obtained directly from the observed data *Y*(*t*) and ∆*H*^+^(*t*) by the equality $\mathbbm{E}\left[{I}_T(t)\right]={\sum}_{i=1}^t\Delta  Y(i)\cdotp {\overset{\sim }{\pi}}_{t-i}$, where ${\overset{\sim }{\pi}}_t$ was the probability that a tested infectious individual remained contagious (i.e. not hospitalized or recovered) for more than *t* days. Since none of the untested infectious individuals were hospitalized, the expected total number of infectious individuals at day *t* was calculated by assuming that the proportion of tested individuals *ρ* is known. For simplicity, we assumed that hospitalization takes place on the same day of appearance of symptoms, such that expected daily number of infectious individuals could be written as $\mathbbm{E}\left[I(t)\right]={\sum}_{i=1}^t\left(\Delta  Y(i)/\rho -\Delta  {H}^{+}(i)\right)\cdotp {\pi}_{t-i}$, where *π*_*t*_ is the probability that a non-hospitalized infectious individual remains contagious (i.e. did not recover) for more than *t* days. As the number of hospitalized individuals was small compared to the number of infectious individuals this assumption will not influence the results.

Finally, given the approximated values for i). *S*(*t*) and ii). *I*(*t*), we assumed that the number of newly exposed individuals Δ*E*^+^(*t*) at day *t* is Poisson distributed with expectation 0.5(*I*(*t*) + *I*(*t* + 1)) ∙ *β*(*t*) · 0.5(*S*(*t*) + *S*(*t* + 1))/*N*. We averaged out the infectious and susceptible individuals at the start and end of day *t* to improve our estimation. Then, Δ*Y*(*t*), is also Poisson distributed with mean $\mathbbm{E}\left[\Delta Y(t)\right]=\rho {\sum}_{i=0}^t{p}_{t-i}\cdotp \left[I(i)+I\left(i+1\right)\right]\cdotp \beta (i)\cdotp \left[S(i)+S\left(i+1\right)\right]/(4N).$ To estimate *β*(*t*), we fitted for each country a Poisson regression model to the daily increases of tested infected individuals Δ*Y*(*t*), selecting the data from the day of the first COVID-19 fatality in the country. We assumed that *β*(*t*) did not change on the last day of our data and included *ρ* as a separate parameter in the regression analysis. If $\hat{\alpha_i}$ was smaller than −4 (such that $\exp \left(\hat{\alpha_i}\right)<0.02\Big)$, then we set $\hat{\beta}(i)$ equal to $\hat{\beta}\left(i-1\right)$ to overcome numerical issues. Finally, we obtained confidence intervals for the estimates by means of the multivariate delta method. All estimations have been performed in R version 3.6.3.

### Sensitivity analyses

By estimating a daily *β*(*t*) the number of parameters equals the number of observations for each country. Therefore we did not draw inference on the other transition-time parameters as is typically done [2, 5, 21]. Instead, to investigate the robustness of our analysis with respect to the choices for those parameters, we conducted several sensitivity analyses. We changed the mean infectious period from 2.3 to 4.6 days [23] for all individuals, and only for the tested individuals. We changed our mean incubation time to 6.4 days (instead of 5.76 days [10]). Additionally, we tested the influence of choosing a gamma distribution for the infectious period and an exponential distribution for the incubation time. We furthermore investigated the scenario of pre-symptomatic transmission [8, 9, 24]. Finally, we studied the influence of the number of susceptible individuals.

### Governmental interventions

**Fig. 2:**
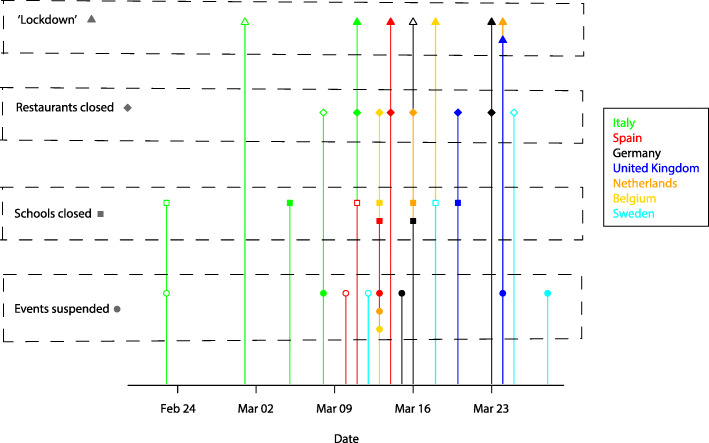
Timing of interventions. Timing of four main governmental interventions (lockdown, restaurants closed, schools closed and events suspended) for the seven European countries considered in this paper. Colors are representative for the countries, while symbols are representative for the measures taken. For each of the considered measures, we have either a filled symbol (measure taken in the whole country), or an empty symbol (measure taken in the ‘red zones’ or at a lower intensity)

In the present study, we focused on four types of governmental interventions that were trying to limit contacts between inhabitants. Figure 2 shows the timing of these four interventions for seven European countries. A full list of governmental interventions can be found in Table S1.

## Results

**Fig. 3:**
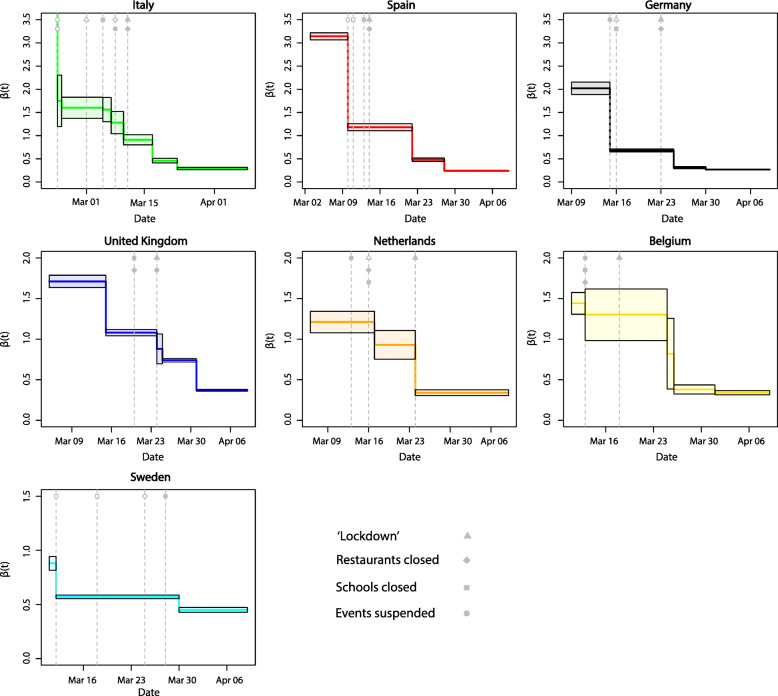
ECR profiles per country. The trend in the point estimates of the ECR $\hat{\beta}(t)$ are presented as colored lines (country specific colors, following the legend in Fig. 2). The black continuous lines represent the 95% confidence intervals of the point estimates. The gray vertical dashed lines indicate the moments when the governmental measures were taken, with the corresponding symbol(s) (same symbols introduced in Fig. 2)

The estimates of the daily ECR for each of the seven countries, together with their 95% confidence intervals, are provided in Fig. 3. The profiles of the corresponding effective reproduction number are presented in Fig. S1.

All countries had a non-increasing profile of the ECR, because of the model restriction, that consisted of a few piecewise constant values. When measures had an immediate effect on the ECR, we expected to see that the times of change points coincided exactly with at least one of the measures. However, due to the variability in the data, it is very likely that a change point was estimated just one (or two) day(s) after (or before) the real date, since daily ECRs that were similar in a period were merged to create the piece-wise constant ECRs. Then, for smaller changes in ECRs, the distance in time between the implementation of measures and the change point could become larger. In the majority of cases, we did indeed see that the drop in the ECR happened within one day of the implementation of at least one of the measures, suggesting that this measure affected the ECR, possibly in combination with measures implemented few days earlier. We have linked the change points in the ECR profile to the measures taken in each country as described next.

In Italy we estimated a large decrease (to 1.60) after the first measures (closure of schools and event ban in the ‘red zone’) were implemented. Another serious decrease (to 1.28) occurred a day after the ‘red zone’ went into a full lockdown and events were banned in the whole country. A similar decrease (to 0.91) was found, in correspondence with the ordering of a nationwide lockdown. However, we estimated a further serious decrease (to 0.46), almost a week after the lockdown was ordered. In this period, Italy enforced the lockdown with increased police forces, assisted by the army (Appendix A). For Spain, we estimated two large decreases. The first change happened within a day since the banning of events and closure of schools in the ‘red zone’ (3.14→1.18). The second effect (to 0.48) became visible a week after the whole country went in lockdown. As in Italy, the Spanish government seriously increased the amount of police forces on the street days after ordering the lockdown. In Germany the first set of official measures of banning events, railway traffic reduction and closure of schools coincided with the first serious decrease in the ECR (2.02→0.68), one day before the ‘red-zone’ Bavaria went in lockdown. Two days after a full lockdown was ordered, we assessed a second change (to 0.31). For the UK, a first decrease in ECR (1.71→1.08) was visible when the UK Chief Medical Officers raised the risk-level to high (Appendix A), a measure that was initially not considered in the present study (Fig. 2). A second decrease in the ECR (to 0.74) was estimated after the lockdown. The third and last decrease (to 0.37) was not traceable to the mentioned four measures but might be attributable to letters sent to 30 million households, containing details on the lockdown, rules and health information. In the Netherlands the ECR decreased (1.21→0.93) on the day after the schools and the restaurants were closed. A more serious drop (to 0.34) became visible after gatherings were officially banned. In Belgium, the estimated ECR did first decrease (1.44→1.30) after events were banned and schools and restaurants were closed. More than a week after the lockdown was ordered, a serious decrease was estimated (to 0.34). In Sweden, a decrease in the ECR (0.88→0.57) became visible when the Swedish government warned its citizens for the first time and banned large events. After more restrictive measures (banning of smaller events) were ordered, a small decrease (to 0.45) was estimated. The dates of the change points in the ECR profile and the measures that seemed to be associated with it are summarized in Table 1. A comparison of the consequences of different measures across countries is presented in the Discussion section.

**Table 1:** Overview of the most significant changes in the ECR profiles. For each country we summarize which governmental measures were taken in close proximity to the changes estimated in the ECR $\hat{\beta}$. The numerical values of the $\hat{\beta}$ before and after the measures are presented together with the relative and absolute change

Country	Date	Measures	ECR (*β*) change	Relative change	Absolute change
Italy	02–23	Closure of schools + banning events in red zone	9.14 → 1.60	0.83	7.54
	03–07	Lockdown in red zone	1.56 → 1.28	0.18	0.28
	03–10	Full lockdown	1.28 → 0.91	0.29	0.37
	03–17	None (enforced police force)	0.91 → 0.46	0.49	0.45
Spain	03–10	Closure of schools + banning events in red zone	3.14 → 1.18	0.62	1.96
	03–22	None (enforced police force)	1.18 → 0.48	0.59	0.70
Germany	03–15	Closure of schools + event banning (+ railway reduction)	2.02 → 0.68	0.66	1.34
	03–25	Lockdown	0.68 → 0.31	0.54	0.37
UK	03–15	None (increased to high risk level)	1.71 → 1.08	0.37	0.63
	03–24	Lockdown	1.08 → 0.74	0.32	0.34
	03–31	None (information to public)	0.74 → 0.37	0.50	0.37
Netherlands	03–17	Closure of schools + restaurants (+ request to stay inside)	1.21 → 0.93	0.23	0.28
	03–24	Lockdown (fines + enforced police force)	0.93 → 0.34	0.63	0.59
Belgium	03–13	Closure of schools + restaurants + banning events	1.44 → 1.30	0.10	0.14
	03–25	None (lockdown extended)	1.30 → 0.34	0.74	0.96
Sweden	03–12	Banning events + warnings to public	0.88 → 0.57	0.35	0.21
	03–29	Stricter measures banning events	0.57 → 0.45	0.21	0.12

In Table 2 we reported the estimated ECR before any interventions were implemented (${\hat{\beta}}_{start}\Big)$, and the final ECR after all interventions were taken (${\hat{\beta}}_{end}\Big)$. The ${\hat{\beta}}_{start}$ varied largely across countries, from 0.88 for Sweden to 9.03 for Italy. However, the variability in ${\hat{\beta}}_{end}$ is much smaller ([0.24, 0.45]). We estimated similar ECR in Italy (0.29), Spain (0.24), and Germany (0.27), while the ECR in the Netherlands (0.34), Belgium (0.35) and the UK (0.37) were somewhat higher. The ECR in Sweden (0.45), where the least rigorous measures were implemented, seemed to be the highest. The estimated average fraction of tested infectious individuals *ρ* can be found in Table S3.

**Table 2:** ECR change. Estimates of the ECR at the start of the considered time window, ${\hat{\beta}}_{start}$, and the rate at the end of the window ${\hat{\beta}}_{end}$ with corresponding standard errors

	${\hat{\boldsymbol{\beta}}}_{\boldsymbol{start}}$	${\hat{\boldsymbol{\beta}}}_{\boldsymbol{end}}$
Italy	9.031 (0.333)	0.290 (0.010)
Spain	3.266 (0.038)	0.240 (0.004)
Germany	2.017 (0.068)	0.271 (0.016)
UK	1.688 (0.037)	0.370 (0.005)
Netherlands	1.212 (0.067)	0.340 (0.019)
Belgium	1.440 (0.057)	0.340 (0.012)
Sweden	0.880 (0.032)	0.452 (0.011)

**Fig. 4:**
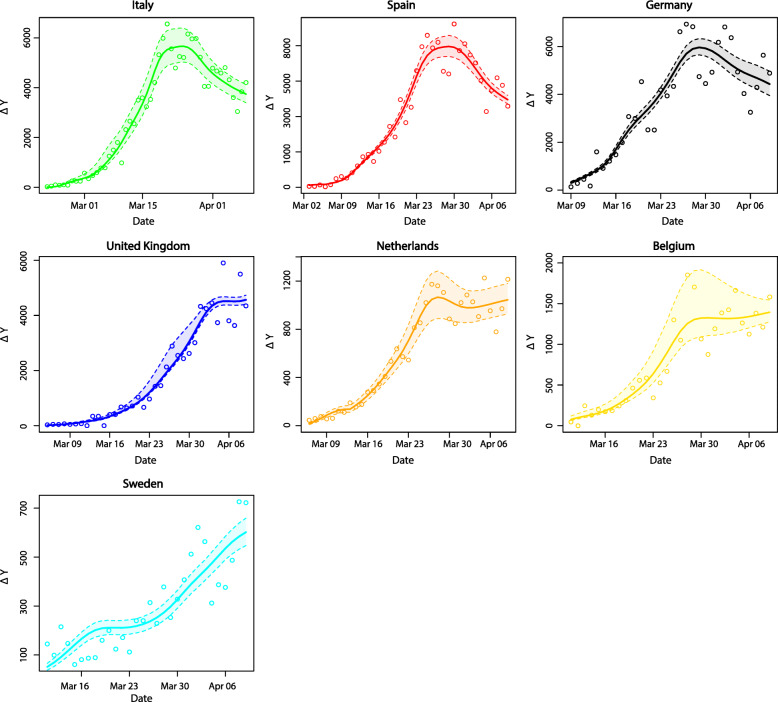
Observed and estimated number of daily new confirmed infections. The daily counts ΔY(t) (points) are presented together with the estimated expected daily new counts from our Poisson regression model (continuous line). Colors are again representative of countries (following the legend in Fig. 2). Furthermore, 95% prediction intervals are presented based on 10,000 simulations assuming multivariate normality of the maximum-likelihood estimates of $\hat{\upalpha_{\mathrm{i}}}$

In Fig. 4, the daily observed number of new confirmed infections Δ*Y*(*t*) (the points in the graph) are shown together with the fit of our Poisson regression model (the continuous lines) and their corresponding 95% prediction intervals. The results of the sensitivity analyses can be found in Figs. S2-S8, demonstrating that the obtained ECR profiles were robust against violation of our assumptions.

## Discussion

In this study we have extended the SEIR model to allow for general transition-time distributions and developed an estimation strategy in which we first retrieved the latent number of infectious and susceptible individuals per day, and subsequently estimated the ECR per day. This way, we did not make a priori assumptions on consistency across countries and delays in the effect of governmental interventions. The change points found in Germany (15/3 and 25/3) do closely agree with the change points found elsewhere (15/3 and 22/3) [5]. Our findings show that change points in the daily ECRs overlap with moments of governmental interventions. Overall, the first set of measures taken by the governments seems to have an immediate (although heterogeneous) effect. This first set of measures typically included the closure of schools and daycares, as well as banning events. Implementing a full lockdown after the first set of measures, either locally or nationally, seemed to coincide with changes in the ECR, but in several countries only when this was enforced with increased police surveillance. Although the effect of closing schools and banning events has been demonstrated in previous studies [1, 4], our findings suggested a stronger (combined) effect of the first set of measures than found before. On the contrary, these studies estimated more serious effects of a gathering bans, lockdowns, and closure of bars and restaurants [1, 4]. These differences may be explained by the additional model restrictions (constant contact rates, country homogeneous-effect sizes, and fixed delay in the effects) that these studies implemented. Therefore, they could not determine whether the effect of the full lockdown did change over time as we estimated. Our observation was in line with the Google mobility data (Appendix F) that suggested that mobility did further reduce in the days after the ordering of the lockdown in Italy, Spain and (less) in the UK and Belgium. The closure of bars and restaurants frequently coincided with the moment of lockdown (Italy, Spain and Germany) or with closing schools (UK, Netherlands, Belgium), thus it is difficult to distinguish the effect of the different measures. In Italy and Spain, the combined measures had only limited influence on the contact rate, and in Sweden national restrictions on restaurant and bars had no effect. So, it seemed unlikely that the closure of bars and restaurants had an additional effect (to the previous measures taken) on the ECR.

Our findings strongly suggested that the ECRs in Italy, Spain, and Germany were very similar after all measures were implemented. This was unexpected since more restrictive measures were taken in Italy and Spain, but it was consistent with other findings [1]. The final ECRs for the Netherlands, Belgium and the UK were also comparable but somewhat higher. The difference between the two groups was not the result of lower compliance to lockdown restrictions, since the Google mobility data showed similar decreases in activity in Belgium and the UK as observed in Spain and Italy. We did therefore conclude that gathering bans (as ordered in Germany and the Netherlands) were as effective as full lockdowns (as implemented in Italy, Spain, UK and Belgium). The value of the limiting ECR level for COVID-19 seems to characterize a society in which citizens only have interactions for essential needs (e.g. grocery shopping and travel of healthcare workers). It is important to observe that despite the fact that the ECRs of the countries were comparable after all the interventions, the ECRs did seriously differ in the period before interventions were implemented. This suggests that the different interventions did not have the same (absolute and relative) effect across countries, as assumed in previous studies [1, 4]. The initial ECR in the UK, Belgium and the Netherlands were similar suggesting the same order of daily contacts which is in line with previous research on social contacts [25]. The initial estimated ECR in Italy should be dealt with caution as the daily increase in confirmed cases before the first death consist of many zeros.

Several limitations of our study need to be addressed. First, our method did not provide estimates of effect sizes for the different measures, contrarily to what is done in previous papers [1, 4], and therefore our results may seem more exploratory. We explicitly made this choice, since interventions were often taken simultaneously and there was no information about the delay on the effect of various measures. All measures try to directly lower the ECR, but the success highly depends on the compliance of the citizens [26]. This was supported by the Google data, showing that reductions in mobility do not align directly with implemented measures. We did not take into account the mixed population (e.g. age groups), but rather tried to estimate the ECR as a country-specific average value. Effect heterogeneity of interventions might thus be attributed to country-specific demographics. Quantification of this effect heterogeneity and further explanation was outside the scope of this study. A second limitation of our approach is the implemented restriction, imposing non-increasing ECRs. This choice was motivated to avoid additional oscillations in the parameter estimate due to the large variability present in the data (shown by the observations outside the prediction intervals in Fig. 4), possibly a result of delays in testing and reporting. Thirdly, our analysis was based on a few assumptions (although supported by other research), like the choice of distributions for the incubation and infectious periods, the number of susceptible individuals and absence of pre-symptomatic transmission. The influence of our modelling assumptions was investigated in the sensitivity analysis (Appendix D) and did not seriously shift the change points. Also, we did split infectious individuals into *I*_*NT*_ and *I*_*T*_ without any link between these classes. The alternative is that everyone would pass the *I*_*NT*_ state before entering *I*_*T*_, but then we could have all kinds of delays in between depending on the capacity of testing. We believe that this is the role of *E*(*t*). Lastly, we assumed that the fraction of tested infectious individuals *ρ* was unknown but constant, while this changed with test policies within countries. It is important to mention that a change in *ρ* over timescales larger than 11 days will not have a serious effect on our estimation (Appendix E). Furthermore, the sensitivity analysis showed that the percentage of tested individuals did not influence the shape of the ECR profile. When data on the daily number of tests and the percentage of positive test would be available, this could help improve the estimation of *ρ*, but this type of information is not always provided by each country.

The main strength of our work is the data-oriented approach: we focused on the information present in the data and modelled it with an extended version of a well-known epidemic disease model, based on a limited number of assumptions. The data demonstrated essential change points in ECR and the sensitivity analysis demonstrated that our assumptions have little influence, making the ECR profile trustworthy. This solid ECR profile could then be connected to moments at which governments implemented measures, evaluating direct and delayed effects. We believe that such approaches are more valuable than the approaches used so far, since the data collection does not satisfy assumptions that are normally valid in other areas of epidemiological research.

We did not build a model that related interventions to the ECR. Therefore, our approach cannot be used to predict future evolution of the ECR. Although we estimated that the first set of governmental interventions had an immediate causal effect, the causal pathway of the first measures remains unclear. This set of measures might have made citizens realize the severity of the situation, which consequently made them reduce their social activity [27]. This would confound the effect estimates of the physical constraints introduced by the interventions and should thus not be overlooked. When governments would have decided to relax these interventions, the ECR might have increased both as a direct result of the vanishing physical restrictions, and indirectly via the relaxation in the behavior of citizens. As a result, the ECR before the intervention was implemented can seriously differ from the ECR after the same intervention is lifted again, this complexity cannot be captured with simple modelling. Prediction studies for exit strategies presenting counterfactual scenarios [1, 28–31] should therefore be dealt with caution. Our methodology can also be used to study the effect of the relaxations, but one should adjust the monotonicity assumption by assuming a non-decreasing ECR.

## Conclusions

With our approach and the data of multiple European countries, we have been able to estimate country-specific change points in the ECR without incorporating a priori information on the governmental interventions. Thereafter, we compared the change points in the ECR profiles with the time points measures were taken. An overview of this comparative study is presented in Table 3. In this comparison, we have showed an immediate influence of banning events and closing schools on the spread of the virus, and a somewhat delayed effect of the full lockdown. The latter illustrates once more that effective intervention depends on the compliance of civilians. Closing bars and restaurants, in addition to the initial measures, seemed to have only a limited effect instead.

**Table 3:**
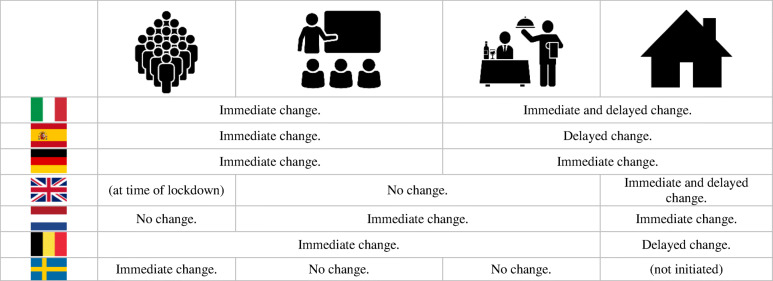
Overview intervention moment and ECR change comparison. Summary of observed ECR profile (see Figure 3) at times of (joint) interventions (event ban, school closure, bar and pub closure, lockdown). ECR did not, dropped immediately or delayed or dropped both immediately and delayed

## Supplementary Information

**Additional file 1.** Supplementary_Postetal_How did governmental interventions affect the spread of COVID-19 in European countries_BMC_vDec2020.pdf– Supplementary Appendix – A: Data sources governmental interventions, B: Discrete time Kolmogorov equations, C: Note on the effective reproduction number, D: Results sensitivity analysis, E: Note on the fraction infected tested and F: an overview of the Google mobility data.

## Data Availability

Data used in this study is obtained from publicly available sources that are referred to in the main text and appendices.

## Abbreviations

SARS-CoV-2: Severe acute respiratory syndrome coronavirus 2
ECR: Effective-contact rate
SEIR: Susceptible-Exposed-Infectious-Removed
COVID-19: Coronavirus Disease 2019
UK: United Kingdom
S: Susceptible
E: Exposed
I: Infectious
R: Removed
