# Supplementary material for: How did governmental interventions affect the spread of COVID-19 in European countries?
Source: BMC Public Health. 2021 Feb 26;21:411. doi: 10.1186/s12889-021-10257-2 (PMC7908011; doi:10.1186/s12889-021-10257-2)
Supplement: Supplementary file 1 — Additional file 1. Supplementary_Postetal_How did governmental interventions affect the spread of COVID-19 in European countries_BMC_vDec2020.pdf– Supplementary Appendix – A: Data sources governmental interventions, B: Discrete time Kolmogorov equations, C: Note on the effective reproduction number, D: Results sensitivity analysis, E: Note on the fraction infected tested and F: an overview of the Google mobility data. [file 12889_2021_10257_MOESM1_ESM.pdf]

Supplementary Appendix for

How did governmental interventions affect the spread of COVID-19 in European countries?

Richard AJ Post, Marta Regis, Zhuozhao Zhan, Edwin R van den Heuvel

A Governmental interventions and data sources

The governmental measures presented in the results section can be found in Table S1. Per country the date the measure was announced (DA) and the date on which the measure can influence the effective-contact rate (DS) are stated. Interventions were often implemented during press conferences in the evening. In that case we decided to let the interventions influence the effective-contact rate from the next day onwards. Furthermore, the sources from where this information is obtained are presented. The data sources for the hospitalization data are presented in Table S2. The hospitalization data was not available for Sweden, but the intensive care unit (ICU) occupation was recorded. We used the (constant) ratio between the Netherlands and Belgium between the hospitalization numbers and the ICU numbers to estimate the hospitalization numbers in Sweden.

	**Intervention**	**DA**	**DS**	**Source**
NL	Events with >100 participants banned<br>Work from home when possible	12 /3	13 /3	https://www.rijksoverheid.nl/onderwerpen/coronavirus-covid-19/coronavirus-beeld-en-video/videos-persconferenties
	Schools and daycares closed<br>Bars and restaurants closed<br>Work from home encouraged	15 /3	16 /3	" "
	Gathering >3 forbidden<br>Fines introduced	23 /3	24 /3	" "
DE	Stricter border control<br>Events ban		15 /3	https://www.deutschland.de/en/news/coronavirus-in-germany-informations
	Schools and daycares closed<br>Railways reduce traffic	15 /3	16 /3	
	Lockdown Bavaria	15 /3	16 /3	
	Gatherings>2 forbidden<br>Bars and restaurants closed	22 /3	23 /3	" "
IT	Schools closed Lombardy<br>Events banned Lombardy		23 /3	http://www.governo.it/it/coronavirus-normativa
	Lockdown Lombardy		1 /3	
	Schools closed	4 /3	5 /3	" "
	Events banned<br>Restrictions bars and restaurants		8 /3	" "
	Lockdown Italy<br>Bars and restaurants closed	10 /3	11 /3	" "
	Army assists police forces		19 /3	https://edition.cnn.com/2020/03/20/europe/italy-military-coronavirus-intl/index.html
	Non-essential production halted	21 /3	22/3	https://www.lavanguardia.com/politica/20200328/48136764563/gobierno-trabajadores-servicios-no-esenciales-30-de-marzo-9-de-abril-semana-santa-pedro-sanchez-coronavirus.html
	Higher fines for violation lockdown rules		24/3	https://www.thelocal.it/20200324/italy-announces-fines-of-up-to-3000-for-breaking-quarantine-rules
ES	Events banned, Madrid		10/3	https://elpais.com/cultura/2020-03-11/coronavirus-el-mundo-de-la-cultura-pone-en-mayo-sus-esperanzas-tras-el-aluvion-de-cancelaciones.html
	Schools closed, Madrid		11 /3	https://english.elpais.com/society/2020-03-09/madrid-basque-city-close-schools-as-coronavirus-continues-spread-in-spain.html
	Schools closed	12 /3	13 /3	https://elpais.com/sociedad/2020-03-12/suspendidas-las-clases-en-todos-los-centros-educativos-de-euskadi.html
	Lockdown<br>Bars and restaurants closed	13 /3	14 /3	https://www.theguardian.com/world/2020/mar/14/spain-government-set-to-order-nationwide-coronavirus-lockdown
	Police force supervise stricter		23 /3	https://www.eldiario.es/politica/FyCSE-realizado-propuestas-sancion-respetar_0_1008599690.html
	Non-essential workers should stay home	28 /3	30 /3	https://www.lavanguardia.com/politica/20200328/48136764563/gobierno-trabajadores-servicios-no-esenciales-30-de-marzo-9-de-abril-semana-santa-pedro-sanchez-coronavirus.html
BE	Schools closed<br>Events banned<br>Restaurants closed	12/3	13 /3	https://www.info-coronavirus.be/en/news/phase-2-maintained-transition-to-the-federal-phase-and-additional-measures/
	Lockdown	17 /3	18 /3	https://web.archive.org/web/20200319160812/https://www.belgium.be/en/news/2020/coronavirus_reinforced_measures
	Closure of borders	20 /3	21 /3	https://www.vrt.be/vrtnws/en/2020/03/20/borders-closed-to-non-essential-travel/
	People arriving at Brussels Airport should be quarantined		25 /3	https://www.vrt.be/vrtnws/en/2020/03/25/people-arriving-in-belgium-quarantined-for-a-fortnight/

	Measures extended		27 /3	https://www.vrt.be/vrtnws/en/2020/03/27/measure-to-curb-the-spread-of-the-novel-coronavirus-to-remain-in/
UK	National risk level raised to high		12 /3	https://www.gov.uk/government/news/covid-19-government-announces-moving-out-of-contain-phase-and-into-delay
	Citizens should avoid non-essential travel	15 /3	16 /3	https://www.bbc.co.uk/news/uk-51917562
	Schools closed<br>Bars and restaurants closed	18 /3	20 /3	https://www.bbc.com/news/uk-51952314<br>https://web.archive.org/web/20200323004800/http://www.legislation.gov.uk/uksi/2020/327/pdfs/uksi_20200327_en.pdf
	Lockdown<br>Events ban	23 /3	24 /3	https://www.gov.uk/government/speeches/pm-address-to-the-nation-on-coronavirus-23-march-2020
	Warning letters send out by government	29 /3	30 /3	https://www.bbc.com/news/uk-politics-52079922
SW	Events >500 banned	11/3	12/3	https://www.gp.se/nyheter/sverige/klart-inga-evenemang-med-fler-%C3%A4n-500-bes%C3%B6kare-1.25224161
	No doctor's certificate needed to stay home	13/3	14/3	https://www.svt.se/nyheter/inrikes/regeringen-kravet-pa-lakarintyg-vid-sjukdom-avskaffas
	Universities and secondary schools closed	17/3	18/3	https://www.svd.se/presstraff-i-dag-med-utbildningsministern
	Restrictions bars and restaurants	24/3	25/3	https://www.svt.se/nyheter/inrikes/nya-forbudet-endast-bordsservering-tillats-pa-restauranger-och-barer
	Events >50 banned	27/3	28/3	https://www.svd.se/stefan-lofven-och-rikspolischefen-haller-presstraff

Table S1 Governmental interventions. Short description of governmental intervention, the date the intervention was announced (DA), the date from which we assumed the measure could affect the ECR (DS), and the source of the information per country. Interventions in grey do not belong to the four groups of measures we focused on in this study.

Italy	http://www.salute.gov.it/imgs/C_17_notizie_4445_0_file.pdf
Spain	https://www.mscbs.gob.es/en/profesionales/saludPublica/ccayes/alertasActual/nCov-China/documentos/Actualizacion_66_COVID-19.pdf
Germany	https://www.rki.de/DE/Home/homepage_node.html
United Kingdom	https://www.gov.uk/government/collections/slides-and-datasets-to-accompany-coronavirus-press-conferences#history
Netherlands	https://www.rivm.nl/coronavirus-covid-19/actueel
Belgium	https://epistat.wiv-isp.be/covid/
Sweden	https://www.folkhalsomyndigheten.se/smittskydd-beredskap/utbrott/aktuella-utbrott/covid-19/bekraftade-fall-i-sverige/

Table S2 Hospitalization data sources.

B Discrete time Kolmogorov equations

The majority of the transition times in the system illustrated in Figure 1 are assumed to follow a Exponential distribution, such that the transitions can be described by continuous-time Kolmogorov equations.

The outflow (and change) of susceptible individuals is described by

$$\Delta S(t) = -I(t)\beta(t)\frac{S(t)}{N}.$$

Consequently, the inflow of exposed individuals is described by

$$\Delta E^{+}(t) = I(t)\beta(t)\frac{S(t)}{N},$$

where $E^{+}(t) = E(t) + I_T(t) + I_{NT}(t) + R_{NT}(t) + R_T(t) + H_T(t)$.

As the incubation time is assumed to follow a Weibull distribution, we cannot describe the outflow of $E(t)$ with a Kolmogorov equation. However, the expected outflow at day t can be given as

$$\mathbb{E}[\Delta I^{+}(t)] = \sum_{i=0}^{t} p_{s-i} \cdot \Delta E^{+}(i),$$

where $I^{+}(t) = E^{+}(t) - E(t)$, p_s is the discretized Weibull probability that a susceptible individual becomes infectious s days after the beginning of the exposure. Similarly, the expected change in exposed individuals equals

$$\mathbb{E}[\Delta E(t) - \Delta I^{+}(t)] = I(t)\beta(t)\frac{S(t)}{N} - \sum_{i=0}^{t} p_{s-i} \cdot \Delta E^{+}(i).$$

A fraction ρ of the expected new number of infectious individuals at day t is tested such that

$\mathbb{E}[\Delta I_T^{+}(t)] = \rho \sum_{i=0}^{t} p_{s-i} \cdot \Delta \mathrm{E}^{+}(i)$ and $\mathbb{E}[\Delta I_{NT}^{+}(t)] = (1-\rho) \sum_{i=0}^{t} p_{s-i} \cdot \Delta \mathrm{E}^{+}(i)$.

The outflow of infectious individuals can again be described with the Kolmogorov equation.

$$\Delta I^{-}(t) = \gamma_I I_{NT}(t) + (\gamma_I + \omega) I_T(t),$$

where $I^{-}(t) = R_{NT}(t) + R_T(t) + H_T(t)$. This outflow equals the sum of the inflow of the hospitalized tested individuals and recovered individuals such that

$$\Delta R_{NT}(t) = \gamma_I I_{NT}(t),$$

$$\Delta H_T(t) = \gamma_I I_T(t) \text{ and}$$

$$\Delta R_T(t) = \gamma_I I_T(t) + \gamma_H H_T(t).$$

C Effective reproduction number

Based on the extended SEIR model presented in Figure 1 (main text) the effective reproduction number is equal to

$$R_e(t) = \left(\left(1-\rho+\rho\left(\frac{\gamma_I}{\gamma_I+\omega}-1\right)\right)\gamma_I^{-1}+\rho\left(\frac{\omega}{\gamma_I+\omega}\right)\omega^{-1}\right)\beta(t)S(t)/N.$$

Under the assumption that $\omega \ll \gamma_I$ or $\omega \approx \gamma_I$ the effective reproduction number simplifies to $\gamma_I^{-1}\beta(t)S(t)/N$. The $\gamma_I^{-1}\beta(t)S(t)/N$ profiles based on our ECR profile estimates and $\gamma_I^{-1} = 2.3$ are presented in Figure S1.

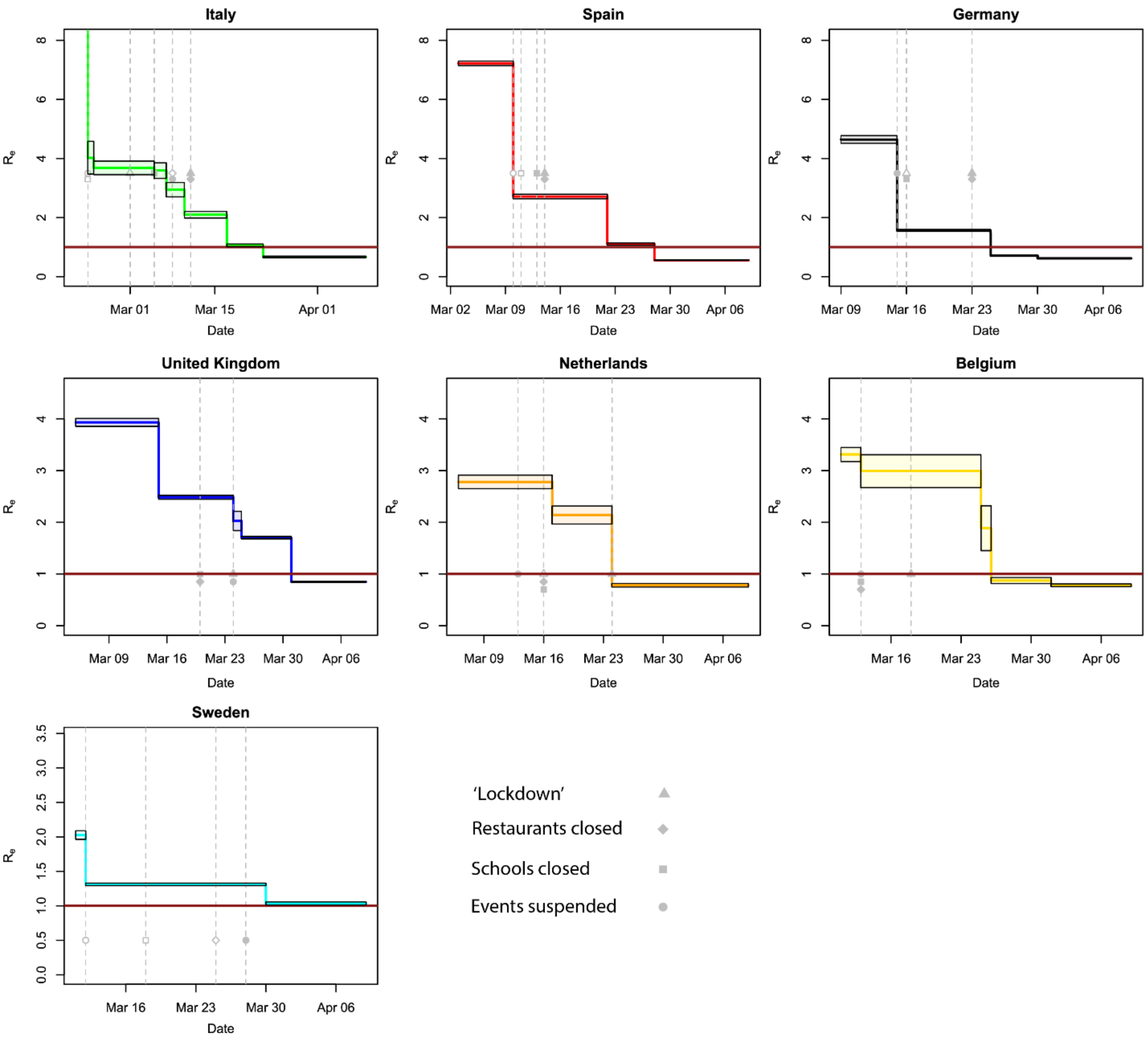

Figure S1 R_e profiles per country. The trend in the point estimates of the $R_e(t)$, assuming an average infectious period of 2.3 days, are presented as colored lines (country specific colors, following the legend in Figure 2). The black continuous lines represent the 95% confidence intervals of the point estimates. The gray vertical dashed lines indicate the moments when the governmental measures were taken, with the corresponding symbol(s) (same symbols introduced in Figure 2).

D Sensitivity analysis

As a reference ECR profile we kept the same settings as in the main analysis, but fixing the fraction of tested individuals to $\rho = 0.1$. Our sensitivity analysis did investigate: *i).* different values of ρ ($\rho = 0.01$), *ii).* different parameters for the distribution of the incubation period, *iii).* an Exponential distribution for the incubation period, *iv).* a Gamma distribution instead of Exponential for the infectious period, *v).* a expected infectious period of 4.6 days instead of 2.3 days, *vi).* a longer expected infectious period for the tested infectious individuals (4.6 days) than for the non-tested infectious individual (2.3 days), *vii).* the possibility of pre-symptomatic transmission[1–3] during which the ECR equals 10% of the ECR after the incubation time and *viii).* a susceptible population consisting of only 10% of the country's population.

In the case of *vii* the estimation equation (1) should be adapted to

$$\mathbb{E}[\Delta Y(t)] = \sum\nolimits_{i=0}^{t} p_{t-i} \cdot \rho \cdot (I(i) + 0.1E(i)) \cdot \beta(i) \cdot S(i)/N.$$

Change *vii* resulted in a higher number of infectious individuals, and thus in a decreased absolute value of $\beta(t)$, but the change points did not shift. Such a change in the absolute value of the ECR is also observed when assuming that the expected length of the infectious periods is longer, case *v*. A slightly higher value for the ECR was estimated in the case of a smaller susceptible population, case *viii*. It is important to notice that the last drop in Italy's profile is still estimated in the latter case and can thus not be explained by a smaller susceptible population (e.g. only in the 'red-zone').

A small shift in the change points was observed when assuming an Exponential incubation time instead of the Weibull distribution, case *iii*. Such a small shift in the change points is also observed when we vary the parameters of the incubation time to the parameters obtained in the Backer et al. study[4] ($n = 88$, mean is 6.40), case *ii*. The parameter for the incubation time used in the main analyses was taken from their preliminary results ($n = 33$) and did align better with other studies reporting a mean incubation time equal to 5.6[5], 5.5[6], 5.2[2] and 5.2[3] respectively. If the mean incubation time was longer, then the change points would be delayed, while the opposite would happen if the mean was shorter. A slightly shorter incubation time could thus explain the reason why we estimate change points the day before measures were implemented, as happens for Italy, Germany, the UK and Spain. However, the latter point could also be explained by the variability in the data as well as the definition of the moment the measure was implemented.

For case *vi* the approximation of $I(t)$ should be adapted to

$$\mathbb{E}[I(i)] = \sum\nolimits_{k=1}^{i} \big(\Delta Y(k) - \Delta H^{+}(k)\big) \cdot \pi_{T,i-k} + \left(\Delta Y(k) \cdot \frac{(1-\rho)}{\rho}\right) \cdot \pi_{NT,i-k},$$

where $\pi_{T,t}$ and $\pi_{NT,t}$ equal the probability that the infectious period is longer than t days for tested and non-tested individuals respectively. The estimated ECR profile will depend on the average infectious period that is mainly determined by the non-tested individuals (90%) as a result of which the ECR profile is very similar to the base case (where all individuals have an expected infectious period of 2.3 days). In the case of a Gamma distribution instead of an Exponential distribution for the infectious period or a $\rho = 0.01$ instead of $\rho = 0.1$, case *iv* and *i*, the ECR profile was also similar to the base case.

The ECR profiles per country for the different sensitivity scenarios are presented together with the ECR profile under the assumptions of the main analysis, but fixing $\rho = 0.1$ in Figures S2-S8. For each country the reference curve is very similar to the one presented in the main results, but since the ρ was taken fixed we could perform the regression on all available ΔY data in the considered time window. For Germany and the United Kingdom this results in an uninformative decrease in the last two days.

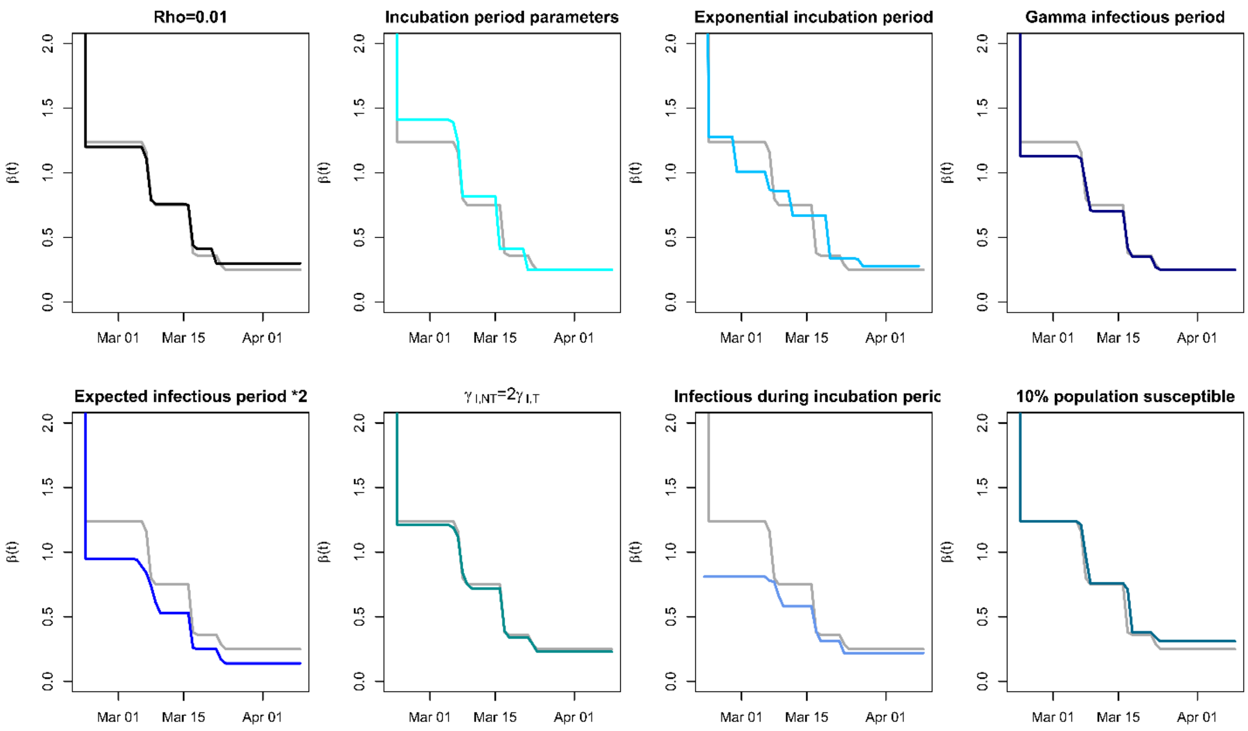

Figure S2: Sensitivity analysis Italy. The ECR profile estimates under the assumptions of the different sensitivity analysis scenarios. The $\rho = 0.1$ analysis is presented as reference curve (grey) in each plot.

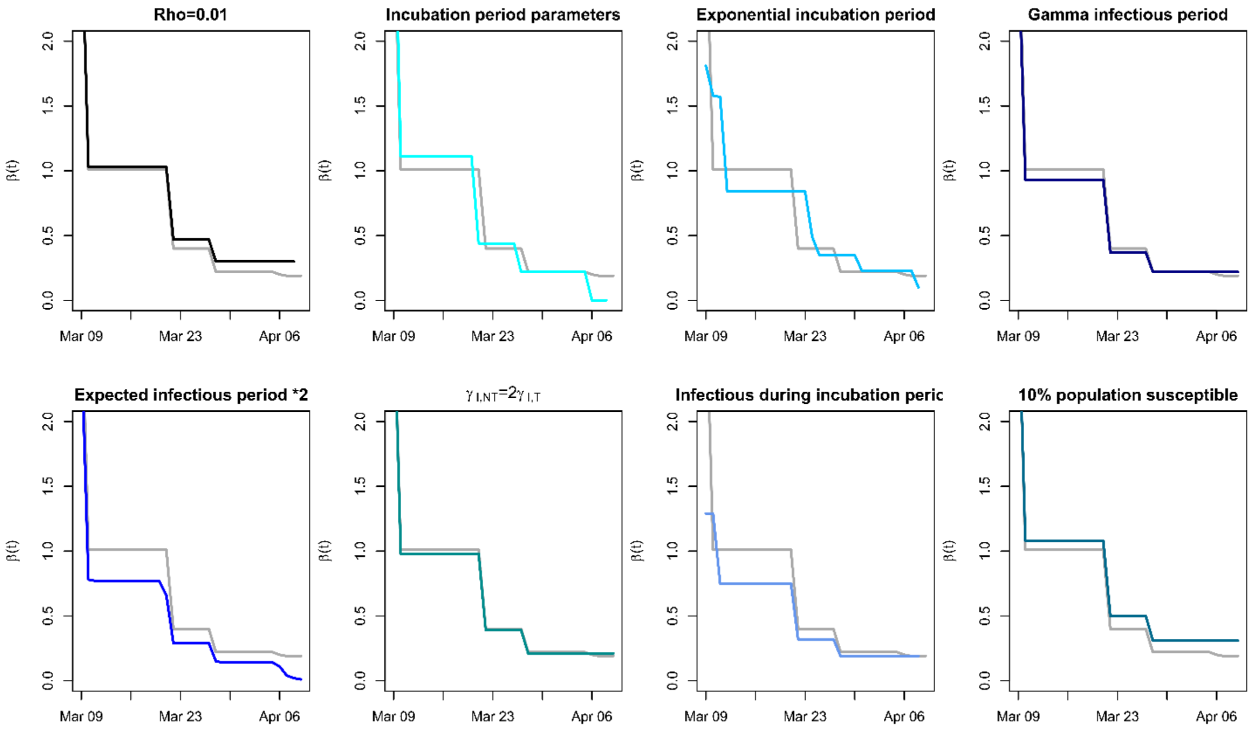

Figure S3: Sensitivity analysis Spain. The ECR profile estimates under the assumptions of the different sensitivity analysis scenarios. The $\rho = 0.1$ analysis is presented as reference curve (grey) in each plot.

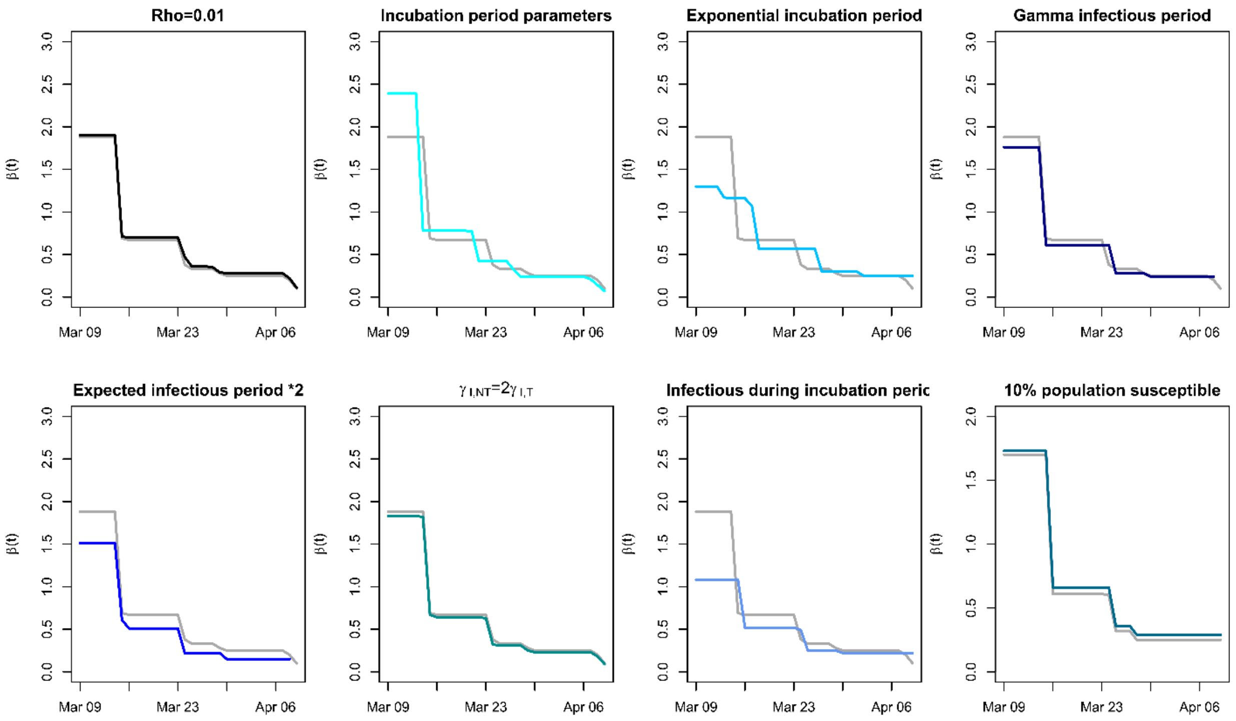

Figure S4: Sensitivity analysis Germany. The ECR profile estimates under the assumptions of the different sensitivity analysis scenarios. The $\rho = 0.1$ analysis is presented as reference curve (grey) in each plot.

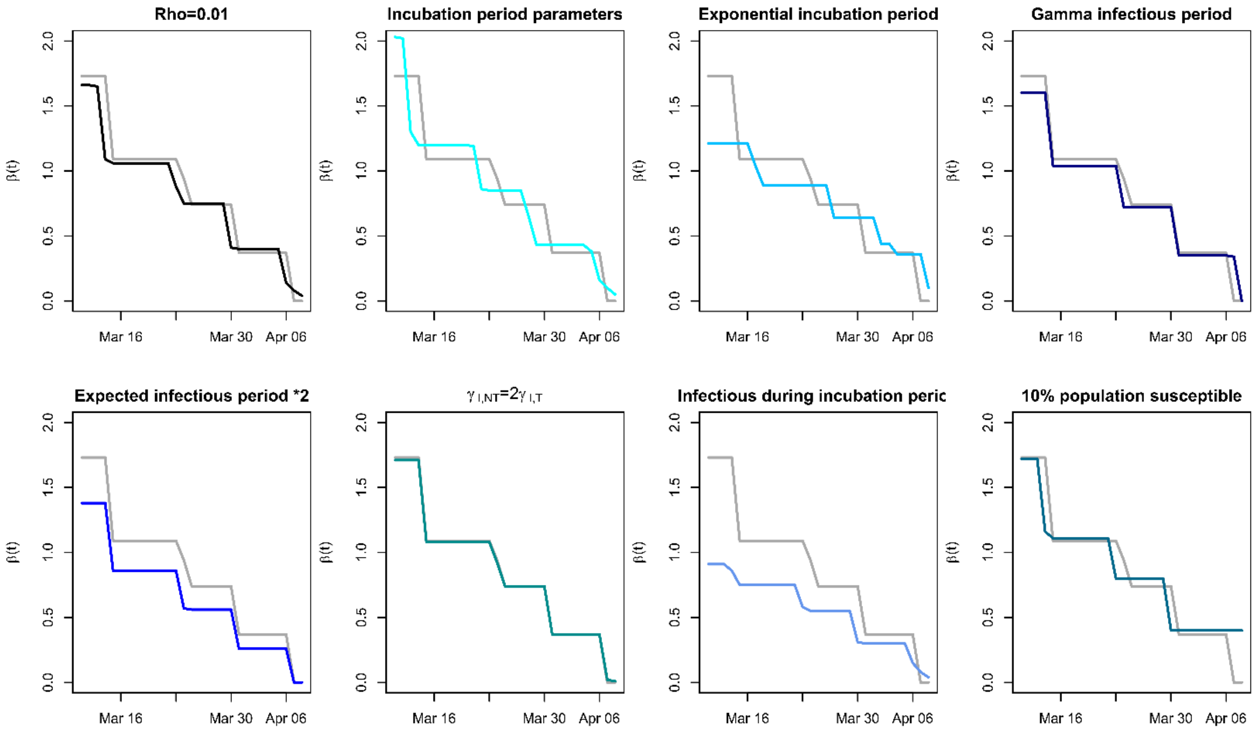

Figure S5: Sensitivity analysis United Kingdom. The ECR profile estimates under the assumptions of the different sensitivity analysis scenarios. The $\rho = 0.1$ analysis is presented as reference curve (grey) in each plot.

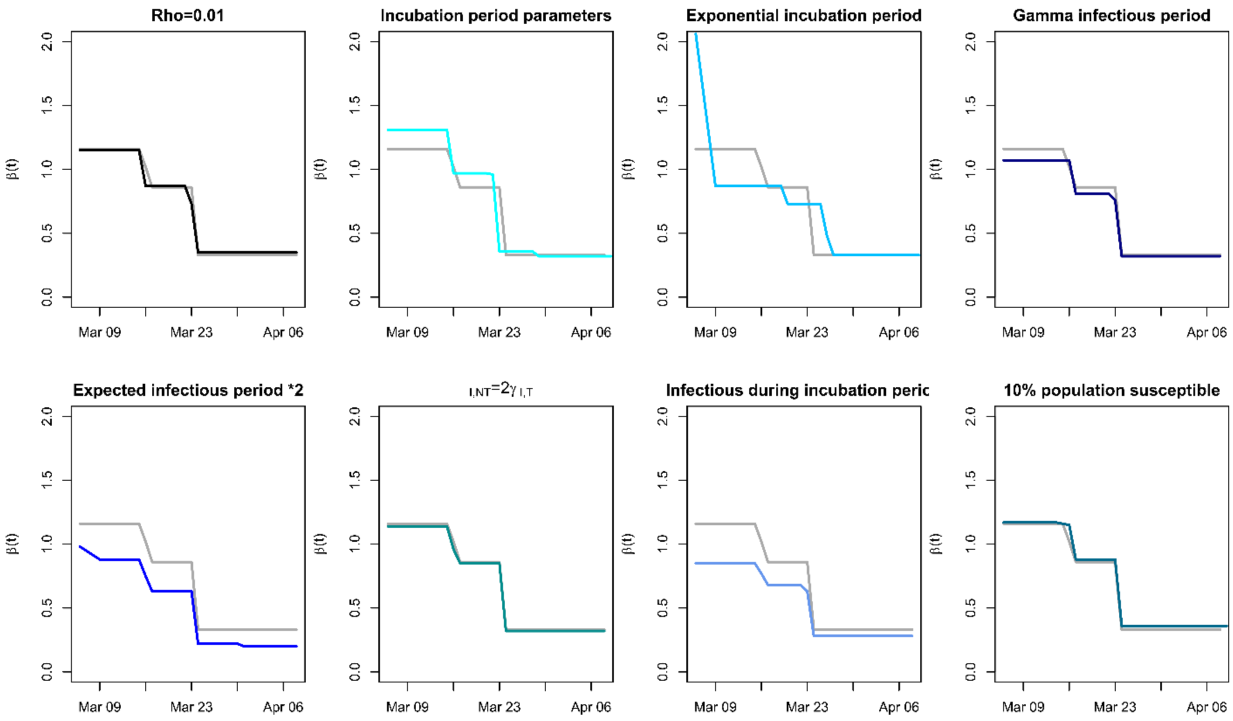

Figure S6: Sensitivity analysis Netherlands. The ECR profile estimates under the assumptions of the different sensitivity analysis scenarios. The $\rho = 0.1$ analysis is presented as reference curve (grey) in each plot.

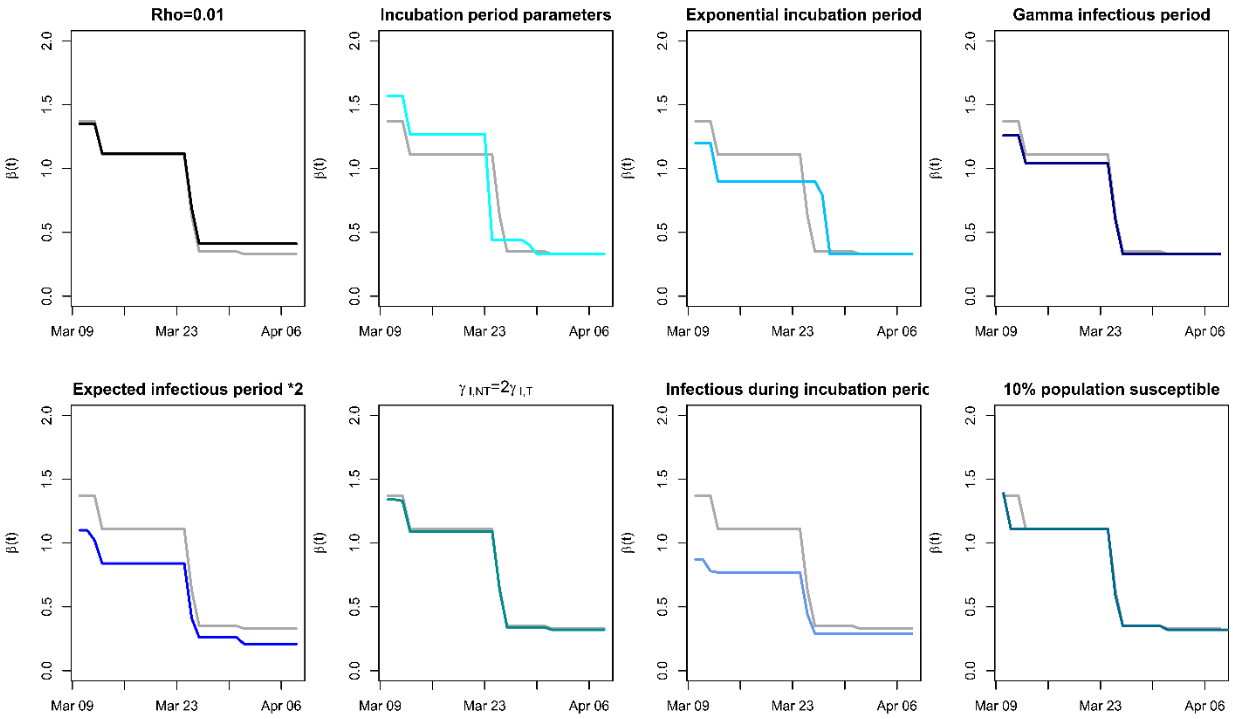

Figure S7: Sensitivity analysis Belgium. The ECR profile estimates under the assumptions of the different sensitivity analysis scenarios. The $\rho = 0.1$ analysis is presented as reference curve (grey) in each plot.

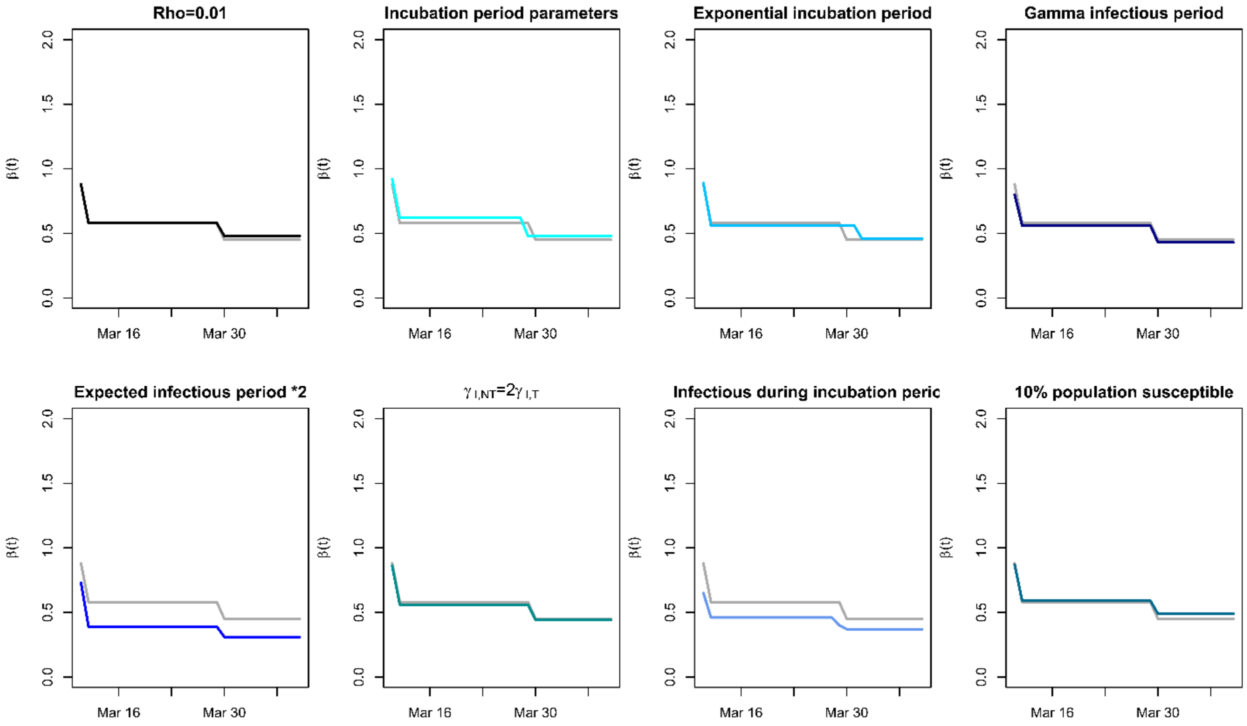

Figure S8: Sensitivity analysis Sweden. The ECR profile estimates under the assumptions of the different sensitivity analysis scenarios. The $\rho = 0.1$ analysis is presented as reference curve (grey) in each plot.

E Fraction of infectious individuals tested

From the sensitivity analysis presented in Appendix B we found that the fraction of tested infectious individuals did not influence the ECR profile. The latter can be explained by the fact that $\mathbb{E}[\Delta Y(t)] \approx \sum_{i=0}^{t} p_{t-i} \cdot I_T(i) \cdot \beta(i) \cdot (\hat{S}(i)/N)$, where only the last term of the product depends on ρ, but at the same time will be close to 1 since in the first phase of the epidemic the vast majority of the population is still susceptible. In the main analysis we did estimate ρ which did thus result in highly variable estimates presented in Table S3. These estimates are highly variable since equation (1), from the main text, can be rewritten as $\mathbb{E}[\Delta Y(t)] = \sum_{i=0}^{t} p_{t-i} \cdot I_T(i) \cdot \beta(i) \cdot \left(1 - \frac{Y(i)+\rho E(i)}{\rho N}\right)$, while at the early stage of the pandemic $Y(t) + \rho E(t) \ll \rho N$, such that the regression becomes independent of ρ.

	$\hat{\rho}$
Italy	0.454 (0.104)
Spain	0.365 (0.047)
Germany	0.780 (0.432)
United Kingdom	0.043 (0.030)
Netherlands	0.207 (0.123)
Belgium	0.369 (0.194)
Sweden	0.028 (0.010)

Table S3 Estimated percentage of tested infectious individuals.

If ρ does vary over time, then

$$\mathbb{E}[\Delta Y(t)] = \rho(t) \sum_{i=0}^{t} p_{t-i} \cdot I(i) \cdot \beta(i) \cdot S(i)/N \text{, and } \mathbb{E}[I(t)] = \sum_{i=1}^{t} \left(\frac{\Delta Y(i)}{\rho(i)} - \Delta H^{+}(i)\right) \cdot \pi_{t-i}.$$

If ρ is reasonably constant over a period of length k such that π_k is small (if $\gamma_I = 1/2.3$, then $\pi_7 = 0.06$), then

$$\mathbb{E}[I(t)] \approx \sum_{i=1}^{t} \left(\frac{\Delta Y(i)}{\rho(t)} - \Delta H^{+}(i)\right) \cdot \pi_{t-i} \text{, and}$$

$$\mathbb{E}[\Delta Y(t)] \approx \rho(t) \sum_{i=0}^{t} \left(\sum_{k=1}^{i} \left(\frac{\Delta Y(k)}{\rho(t)} - \Delta H^{+}(k)\right) \cdot p_{t-i}\pi_{i-k}\right) \cdot \beta(i) \cdot \left(1 - \frac{Y(i) + \rho(t)E(i)}{\rho(t)N}\right).$$

If ρ is still reasonably constant over period of length $l > k$, such that for all a, b such that $\mathrm{a} + \mathrm{b} > \mathrm{l}$, $p_a\pi_b$ is neglectable (e.g. < 0.05), then

$$\mathbb{E}[\Delta Y(t)] \approx \sum_{i=0}^{t} \left(\sum_{k=1}^{i} \left(\Delta Y(k) - \rho(t)\Delta H^{+}(k)\right) \cdot p_{t-i}\pi_{i-k}\right) \cdot \beta(i) \cdot \left(1 - \frac{Y(i) + \rho(t)E(i)}{\rho(t)N}\right).$$

We can assume $\rho(t)\Delta H^{+}(k) \ll \Delta Y(k)$ and at the early stage of the pandemic $Y(t) + \rho(t)E(t) \ll \rho(t)N$ such that

$$\mathbb{E}[\Delta Y(t)] \approx \sum_{i=0}^{t} \left(\sum_{k=1}^{i} \Delta Y(k) \cdot p_{t-i}\pi_{i-k}\right) \cdot \beta(i),$$

and the regression analysis becomes independent of $\rho(t)$.

Based the assumptions made in this study for all $\mathrm{a} + \mathrm{b}$ equal to 12, $p_a\pi_b < 0.05$, while $p_{10}\pi_1 = 0.053$, such that our analysis is non sensitive to time-varying ρ over timescales larger than 11 days.

F Google mobility data

In the discussion we did compare some of our findings with the data on mobility made available by Google[7]. Here the relative decrease of mobility was provided, regarding retail and recreation, grocery and pharmacy, parks, transit stations, workplaces and residential, with respect to the average activity in the period before the COVID-19 outbreak. The fraction of this baseline activity is presented together with the dates of governmental interventions (the same symbols introduced in Figure 2) for each country in Figures S8-S15.

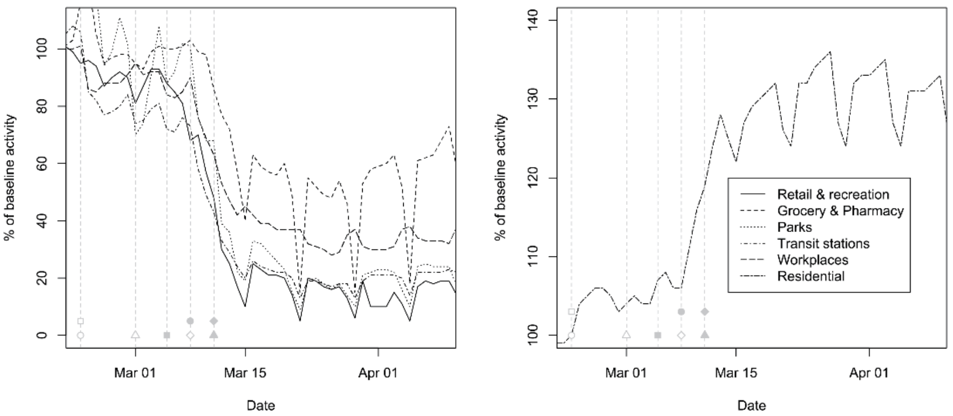

Figure S9: Mobility data Italy. Moments of governmental interventions and the change in mobility activity as a fraction of the baseline (before the COVID-19 outbreak) activity provided by Google[7].

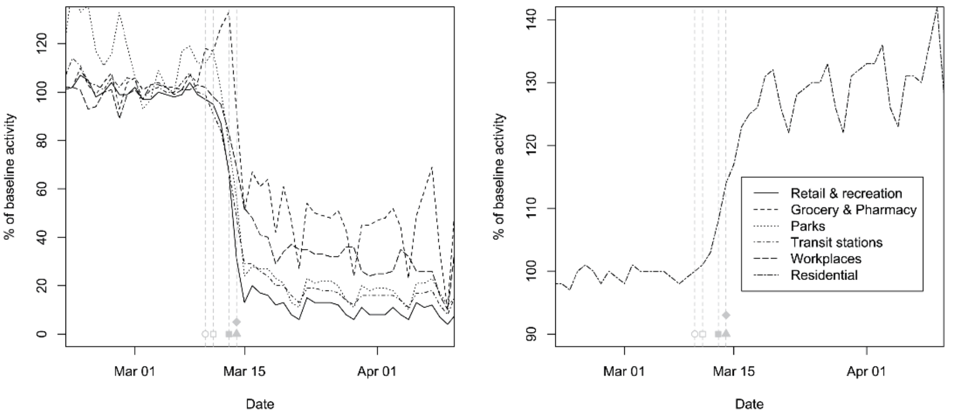

Figure S10: Mobility data Spain. Moments of governmental interventions and the change in mobility activity as a fraction of the baseline (before the COVID-19 outbreak) activity provided by Google[7].

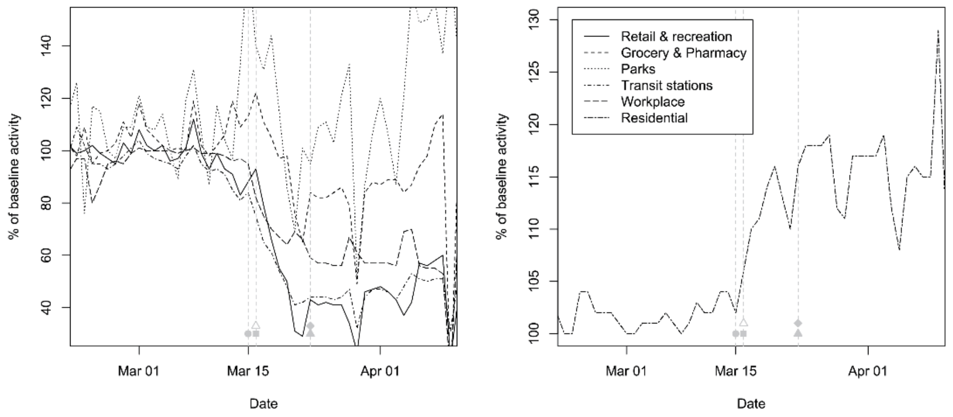

Figure S11: Mobility data Germany. Moments of governmental interventions and the change in mobility activity as a fraction of the baseline (before the COVID-19 outbreak) activity provided by Google[7].

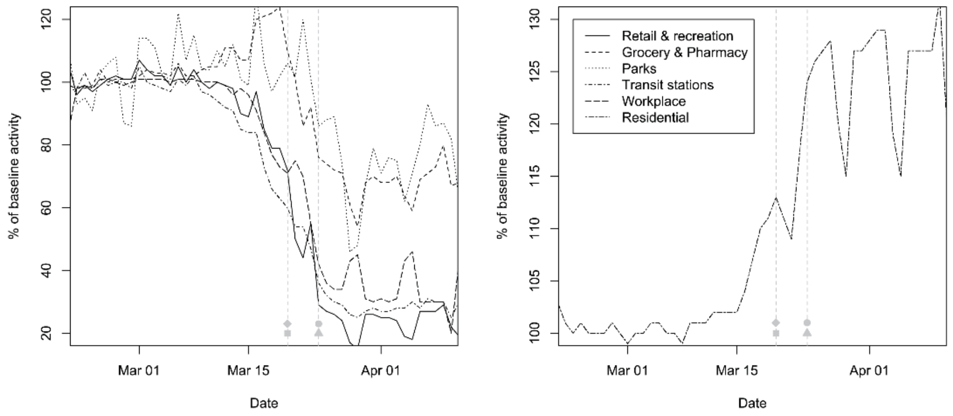

Figure S12: Mobility data United Kingdom. Moments of governmental interventions and the change in mobility activity as a fraction of the baseline (before the COVID-19 outbreak) activity provided by Google[7].

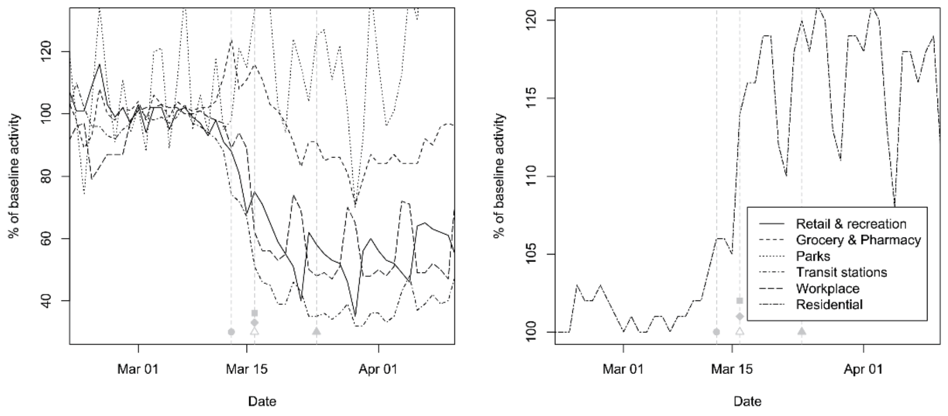

Figure S13: Mobility data Netherlands. Moments of governmental interventions and the change in mobility activity as a fraction of the baseline (before the COVID-19 outbreak) activity provided by Google[7].

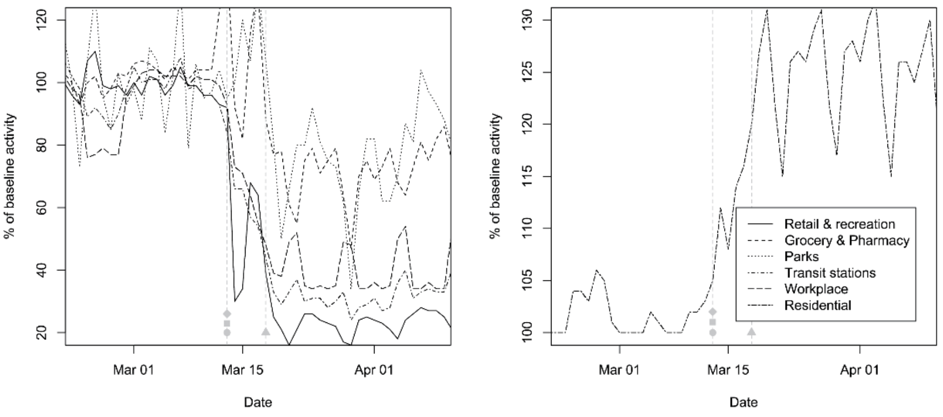

Figure S14: Mobility data Belgium. Moments of governmental interventions and the change in mobility activity as a fraction of the baseline (before the COVID-19 outbreak) activity provided by Google[7].

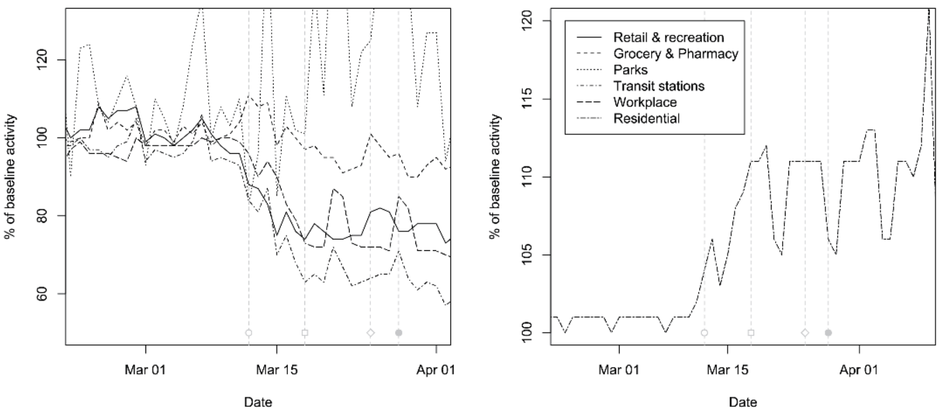

Figure S15: Mobility data Sweden. Moments of governmental interventions and the change in mobility activity as a fraction of the baseline (before the COVID-19 outbreak) activity provided by Google[7].

References

1. Nishiura H, Linton NM, Akhmetzhanov AR. Serial interval of novel coronavirus (COVID-19) infections. Int J Infect Dis. 2020;93:284–6. doi:10.1016/j.ijid.2020.02.060.

2. Li DQ, Guan X, Wu P, Wang X, Cowling DB, Yang B, et al. Early Transmission Dynamics in Wuhan, China, of Novel Coronavirus–Infected Pneumonia. Public Health. 2020;13:1199–207.

3. Zhang J, Litvinova M, Wang W, Wang Y, Deng X, Chen X, et al. Evolving epidemiology and transmission dynamics of coronavirus disease 2019 outside Hubei province, China: a descriptive and modelling study. Lancet Infect Dis. doi:10.1016/S1473-3099(20)30230-9.

4. Backer J, Klinkenberg D, Wallinga J. The incubation period of 2019-nCoV infections among travellers from Wuhan, China. Eurosurveillance. 2020;25:2000062.

5. Linton NM, Kobayashi T, Yang Y, Hayashi K, Akhmetzhanov AR, Jung S, et al. Incubation Period and Other Epidemiological Characteristics of 2019 Novel Coronavirus Infections with Right Truncation: A

Statistical Analysis of Publicly Available Case Data. J Clin Med. 2020;9:538. doi:10.3390/jcm9020538.

6. Lauer SA, Grantz KH, Bi Q, Jones FK, Zheng Q, Meredith HR, et al. The Incubation Period of Coronavirus Disease 2019 (COVID-19) From Publicly Reported Confirmed Cases: Estimation and Application. Ann Intern Med. 2020;172:577–82. doi:10.7326/M20-0504.

7. Google LLC. Google COVID-19 Community Mobility Reports. 2020. google.com/covid19/mobility/. Accessed 5 May 2020.
